# Supplementary material for: Vulnerability and resilience to activity-based anorexia are not sex-dependent
Source: Front Psychiatry. 2026 Oct 2;17:1897501. doi: 10.3389/fpsyt.2026.1897501 (PMC13672643; doi:10.3389/fpsyt.2026.1897501)
Supplement: Supplementary file 1 [file Table1.pdf]

| Figure | Figure Description | Model | Stats Reported |
|---|---|---|---|
| 1A | Change in weight on each day of restriction for males and females | change in % BW ~ expday [as continuous fixed effect] * sex [as factor fixed effect] + (expday \| mouse) [as random effect with change across days (slope) computed for each mouse] | sex x day, $F_{(1,32)}$ = 0.50, p=0.48; sex, $F_{(1,36)}$ = 1.78, p = 0.19 |
| 1E | Change in weight on each day of restriction, males and females of both phenotypes | daily weight loss ~ phenotype [as factor fixed effect] * expday [as continuous fixed effect] * sex [as factor fixed effect ]+ (expday \| mouse) [as random effect with change across days (slope) computed for each mouse] | phenotype x day, $F_{(1,89)}$ = 0.59, p = 0.44; phenotype, $F_{(1,39)}$ = 10.45, p = 0.002; sex, $F_{(1,39)}$ = 1.01, p = 0.32 |
| 2B | Male food intake data on each day of food restriction | consumption ~ phenotype [as factor fixed effect] * expday [as continuous fixed effect] + (expday \| mouse) [radnom effect with change across days (slope) computed for each mouse] | phenotype x day, $F_{(1,14)}$ = 4.23, p = 0.058 |
| 3B1 | Male hourly running during baseline, both phenotypes | wheel turns ~ pheno [as factor fixed effect] * hour [as integer fixed effect] [ + (1 \| mouse [individual mice as random effect, intercept only) | phenotype x hour, $F_{(1,1347)}$ = 1.36, p = 0.24 |
| 3B2 | Male hourly running during ABA, both phenotypes | turns ~ pheno [as factor fixed effect] * hour [as integer fixed effect] + (1 \| mouse) [individual mice as random effect, intercept only] | phenotype x hour, $F_{(1,3147)}$ = 9.70, p = 0.0018 |
